# Vehicle Following Distance Violation Detection with City Surveillance Cameras

**DOI:** 10.3390/s26216815

**Published:** 2026-10-28

**Authors:** Yılmaz Dikilitaş, Ahmet Sayar

**Affiliations:** 1 Department of Computer Engineering, Faculty of Engineering, Kocaeli University, Kocaeli 41001, Türkiye; yilmaz.dikilitas@kocaeli.edu.tr; 2 Department of Artificial Intelligence Engineering, Bahçeşehir University, Istanbul 34353, Türkiye; 3 KOU Teknopark, Bidist, Kocaeli 41275, Türkiye; 4 Department of Computer Engineering, Kocaeli University, Kocaeli 41380, Türkiye

**Keywords:** following distance, time headway, vehicle speed estimation, multi-object tracking, traffic surveillance, surrogate safety measures

## Abstract

Maintaining an adequate following distance reduces the risk of rear-end collisions, but monitoring it with fixed urban surveillance cameras requires image observations to be converted into reliable metric quantities. This study presents and validates a measurement pipeline for following-distance violations from a single fixed camera. Vehicles are detected with YOLO11 and tracked with ByteTrack, BoT-SORT or OC-SORT; the bottom-centre of each bounding box is mapped to a longitudinal road coordinate by a per-lane homography whose along-road scale is calibrated once from loop-meter speeds. Vehicle speed, time headway, front-to-front spacing and time to collision are estimated from the trajectories, and eight violation criteria, including the rule that the distance in metres must not fall below half the speed in km/h, are evaluated together with a probabilistic estimate of decision uncertainty. On 14 public test videos (178 min, 5939 vehicles) with inductive-loop reference speeds, the best configuration (YOLO11-m with BoT-SORT) measured speed with a mean absolute error of 1.04 km/h, time headway with 0.057 s and spacing with 1.14 m, and detected half-speed-rule and 1.8 s headway violations with F1 scores of 0.81 and 0.85; with a headway error model estimated on the calibration video only, 4.5% of the vehicle pairs were identified as uncertain. On the UA-DETRAC benchmark, BoT-SORT achieved the highest HOTA (0.681) and IDF1 (0.790), consistent with its highest pair recall. Headway-based criteria were the most reliable, and the metric scale had to be calibrated on site rather than inferred from road dimensions.

## 1. Introduction

Road traffic crashes remain a major global public health and transportation safety problem despite continuous advances in vehicle technology, intelligent transportation systems, and road infrastructure. According to the World Health Organization (WHO), approximately 1.16 million people die annually as a result of road traffic crashes, while tens of millions experience non-fatal injuries. Road traffic injuries also remain a leading cause of death among children and young adults aged 5–29 years [1]. These figures highlight the continuing need for automated traffic monitoring and preventive safety technologies capable of identifying potentially hazardous driving behavior before it results in a collision.

Rear-end collisions represent an important category of road crashes and are closely associated with car-following behavior, driver reaction time, vehicle speed, and the distance maintained between successive vehicles. Insufficient following distance reduces the time available to react to sudden changes in the motion of a preceding vehicle, and distance headway and driver response behavior are strongly related in dynamic car-following scenarios [2]. Time headway and time to collision are therefore established surrogate indicators of car-following risk [3]. Many traffic codes express the safe following distance as a distance proportional to speed; the rule that the gap in metres should not be smaller than half the speed in km/h, for example, is equivalent to a time headway of 1.8 s.

Existing city surveillance cameras could, in principle, monitor such behavior at scale without dedicated sensors on every vehicle. Modern object detectors and multi-object trackers can follow many vehicles through a video, and recent work has shown that short headways extracted from ordinary traffic cameras are associated with recorded crashes [4]. Turning a fixed monocular camera into a measurement device, however, is harder than detecting and tracking vehicles. Image measurements are affected by perspective, so equal road distances do not correspond to equal pixel distances, and a plane-to-plane homography is needed to map image points onto the road [5]. The resulting road coordinates must then be given a metric scale along the direction of travel, which is the main source of error in camera-based speed measurement [6]. Finally, a violation decision combines several such measurements, so its reliability can only be judged if speed, headway and spacing are each validated against reference values. Without this validation, a following-distance system cannot be distinguished from a visual demonstration.

In this study, we develop and validate a measurement pipeline for following-distance violations from a fixed urban surveillance camera. Vehicles are detected with YOLO11 [7] and tracked with ByteTrack, BoT-SORT and OC-SORT. The bottom-centre of each bounding box is mapped to a metric longitudinal road coordinate by a per-lane homography whose along-road scale is calibrated once from loop-meter speeds and then kept fixed. From the resulting trajectories, we estimate each vehicle’s speed, the time headway and front-to-front spacing of consecutive vehicles in the same lane, and their time to collision, and we evaluate eight violation criteria, including the half-speed rule. All measurements are validated on the public UTFPR urban speed dataset [8], whose speeds come from an inductive-loop meter, and detection and tracking accuracy is reported separately on the UA-DETRAC benchmark [9].

The main contributions of this study are as follows:

- A following-distance measurement pipeline for a fixed monocular camera in which speed, time headway and spacing are estimated in metric road coordinates, with the along-road scale calibrated from measured speeds rather than assumed from lane width.
- A ground-truth validation on 14 public test videos (178 min, 5939 vehicles): speed against inductive-loop measurements (MAE 1.04 km/h), and time headway and spacing against references derived from the dataset annotation (MAE 0.057 s and 1.14 m).
- An evaluation of eight violation criteria with precision, recall and F1, showing that the half-speed rule is equivalent to a 1.8 s time headway (F1 0.81), that a fixed 10 m distance criterion is much less reliable (F1 0.34), and that time-to-collision criteria have too few positives in free-flowing traffic to be assessed, together with a threshold sensitivity analysis and a probabilistic estimate of how many decisions are uncertain.
- A comparison of current detectors and trackers by their detection and tracking accuracy on UA-DETRAC (AP, HOTA, MOTA, IDF1, overall and per weather condition), by their downstream measurement error and by their runtime on a mid-range GPU.

The remainder of this paper is organized as follows. Section 2 reviews related work. Section 3 describes the measurement pipeline and the violation criteria. Section 4 describes the datasets, reference values and metrics and presents the results, and Section 5 discusses them, including their limitations, and concludes this paper.

## 2. Related Work

Camera-based traffic monitoring has moved from feature tracking and background modelling towards deep learning pipelines that detect, track and analyse many vehicles at once. The work relevant to this study falls into four groups: vehicle detection and tracking; camera calibration and speed measurement; headway and trajectory extraction from video; and surrogate safety measures for car following.

### 2.1. Vehicle Detection and Multi-Object Tracking

Single-stage detectors of the YOLO family are the most common choice in traffic applications because they localise and classify vehicles at video rate; this study uses YOLO11 [7]. Detections must be linked over time before speed or spacing can be measured. SORT combines a Kalman-filter motion model with frame-to-frame assignment [10], and Deep SORT adds appearance features to reduce identity switches [11]. ByteTrack also associates low-confidence detections [12], BoT-SORT adds camera-motion compensation and a refined Kalman state [13], and OC-SORT corrects the error accumulated by the motion model during occlusion [14]. Detection and tracking quality are usually reported on benchmarks such as UA-DETRAC [9] with the CLEAR MOT metrics [15], IDF1 [16] and HOTA [17]; we use these in Section 4. Our aim is not a new detector or tracker: a tracker that is adequate for visualisation can still produce large errors once its trajectories are converted into speeds and headways, so we also compare trackers based on their downstream measurement error.

### 2.2. Camera Calibration and Vehicle Speed Measurement

A single image does not preserve metric scale. For a fixed camera observing an approximately planar road, a plane-to-plane homography maps image points to road coordinates [5], but it does not by itself fix a common metric scale in both road directions, so the along-road scale has to be calibrated explicitly. Calibration is the main difficulty in turning surveillance cameras into measurement sensors [6]. Dubská et al. calibrate roadside cameras automatically from the vanishing points of vehicle motion [18], and Revaud and Humenberger estimate speed from uncalibrated CCTV footage [19]. Comparing such methods requires public datasets with ground-truth speeds, of which few exist [6]: BrnoCompSpeed provides speeds from laser gates [20], and the UTFPR dataset speeds from inductive loops [8]. We use the latter because its annotations also give the lane and frame of each vehicle, from which reference headways can be derived.

### 2.3. Headway and Trajectory Extraction from Video

Drone datasets such as highD provide vehicle trajectories from which time headway, distance headway and time to collision are computed for every vehicle [21], but they are recorded from above and avoid the perspective and occlusion problems of a roadside camera. Charouh et al. estimated speed, time headway and following distance from a fixed camera on a highway bridge using a deep learning detector, a homography and a virtual reference line, and they compared them with in-vehicle speed data and manually measured headways for a small number of test runs [22]. St-Aubin et al. developed an automated pipeline that tracks road users in surveillance video and computes surrogate safety measures at scale [23]. Shaer et al. recently extracted short headways and time to collision from ordinary traffic cameras and showed that these indicators are associated with recorded crashes [4]. These studies show that video-based headway indicators are meaningful for safety, but few report the error of the headway or spacing itself against an independent reference, and none that we found evaluate a following-distance rule with precision and recall.

### 2.4. Surrogate Safety Measures for Car Following

Time to collision was introduced by Hayward as a measure of the closeness of a conflict [24]. Vogel showed that time headway and time to collision capture different aspects of car-following risk: short headways indicate potentially dangerous situations even when time to collision is undefined, whereas small time-to-collision values indicate an actual approaching conflict [3]. Mahmud et al. review the thresholds used for these indicators in practice [25]. Because legal following-distance rules are expressed as a distance proportional to speed, they correspond to a time-headway threshold; we therefore evaluate the legal rule together with time-headway, fixed-distance and time-to-collision criteria (Section 3).

### 2.5. Research Gap and Positioning of the Present Study

Detection and tracking, camera calibration and speed measurement, and video-based safety analysis are each well studied [4,6,23]. They are rarely combined into a following-distance measurement whose speed, headway and spacing errors and violation decisions are validated against reference values from a fixed urban camera. This study provides such a validation on a public dataset with loop-meter speeds, reports detection and tracking accuracy on a public benchmark, and quantifies how measurement uncertainty affects the violation decision.

## 3. Materials and Methods

**Figure 1:**
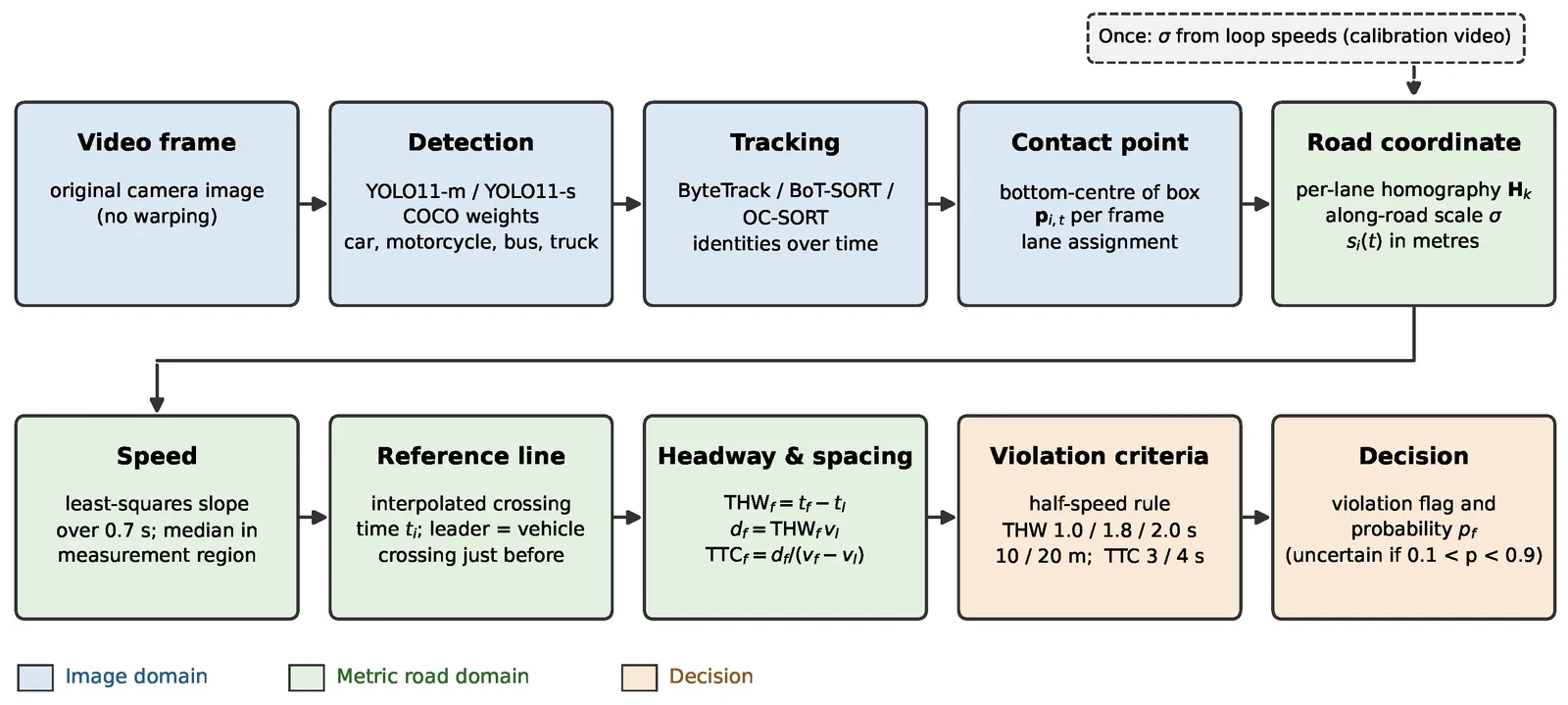
Processing chain of the following-distance measurement framework. Detection and tracking operate on the original image; only the bottom-centre point of each tracked box is mapped to the road plane.

The framework estimates three quantities for every pair of consecutive vehicles in the same lane, a leader *l* and a follower *f*: the speed of each vehicle, the time headway between them, and their longitudinal spacing. A following-distance violation is then a decision on these quantities. The processing chain is shown in Figure 1 and summarized in Table 1. All detection and tracking is performed on the original camera image; only the road contact points of the tracked vehicles are mapped to road coordinates.

**Table 2:** Violation criteria evaluated for each follower.

Criterion	Violation if
Half-speed rule	$d_{f}<v_{f}^{\mathrm{km}/h}/2$ m (Equation (11))
Time headway	${\mathrm{THW}}_{f}<1.0$, 1.8 or 2.0 s
Fixed distance	$d_{f}<10$ m or $d_{f}<20$ m
Time to collision	${\mathrm{TTC}}_{f}<3$ s or <4 s, only when $v_{f}-v_{l}>0.1$ m/s

### 3.1. Vehicle Detection and Tracking

Vehicles are detected in every frame with Ultralytics YOLO11 [7], using the medium model (YOLO11-m) as the main configuration and the small model (YOLO11-s) as a lightweight alternative. Both use pre-trained MS-COCO weights; the COCO classes car, motorcycle, bus and truck are kept, the input resolution is 960 px and the confidence threshold is 0.25 (0.1 for OC-SORT, which applies its own confidence gating). No component is trained or fine-tuned on the evaluation data.

Detections are associated over time with three trackers: ByteTrack [12] and BoT-SORT [13] in their Ultralytics implementations with default parameters, and OC-SORT [14] in the BoxMOT 25.0.0 implementation. All three predict box motion with a Kalman filter but use different state vectors. ByteTrack (Ultralytics) uses the Deep SORT state [11]

(1) $$x={\left(u,\;v,\;\gamma ,\;h,\;\dot{u},\;\dot{v},\;\dot{\gamma },\;\dot{h}\right)}^{T},$$

where (u,v) is the box centre, γ the aspect ratio and *h* the height. BoT-SORT replaces the aspect ratio by the box width,

(2) $$x={\left(u,\;v,\;w,\;h,\;\dot{u},\;\dot{v},\;\dot{w},\;\dot{h}\right)}^{T},$$

and OC-SORT uses the SORT state [10] with box area *a* and aspect ratio *r*,

(3) $$x={\left(u,\;v,\;a,\;r,\;\dot{u},\;\dot{v},\;\dot{a}\right)}^{T}.$$

For each tracked box with corners $\left(x_{1},y_{1}\right)$ and $\left(x_{2},y_{2}\right)$ in the original image, the bottom-centre point

(4) $$p_{i,t}=\left(\frac{x_{1,i,t}+x_{2,i,t}}{2},\;y_{2,i,t}\right)$$

approximates the point where vehicle *i* touches the road at time *t*. Tracks are split at gaps longer than 0.5 s and linearly interpolated over shorter gaps.

### 3.2. Road Coordinates and Along-Road Scale

Each lane *k* has an image quadrilateral and a homography $H_{k}$ that maps image points to a rectified, top-down view of that lane (both provided with the dataset, Section 4):

(5) $$w\left[\begin{array}{c} x^{\prime }\\ y^{\prime }\\ 1 \end{array}\right]=H_{k}\left[\begin{array}{c} x\\ y\\ 1 \end{array}\right],$$

where (x,y) is a point in the original image, $\left(x^{\prime },y^{\prime }\right)$ its position in the rectified view and *w* the homogeneous scale. A track is assigned to the lane whose quadrilateral contains most of its points.

The rectified view preserves straight lines and ratios along the road, but its pixel size along the road is not known in metres and need not equal its pixel size across the road. We therefore use only the longitudinal rectified coordinate $y^{\prime }$ and convert it to metres with a single along-road scale factor σ:

(6) $$s_{i}\left(t\right)=-\sigma \;y_{i}^{\prime }\left(t\right),$$

where *s* increases in the direction of travel. The cross-road coordinate is used only for lane assignment, so no cross-road scale such as the lane width enters any measurement.

**Figure 2:**
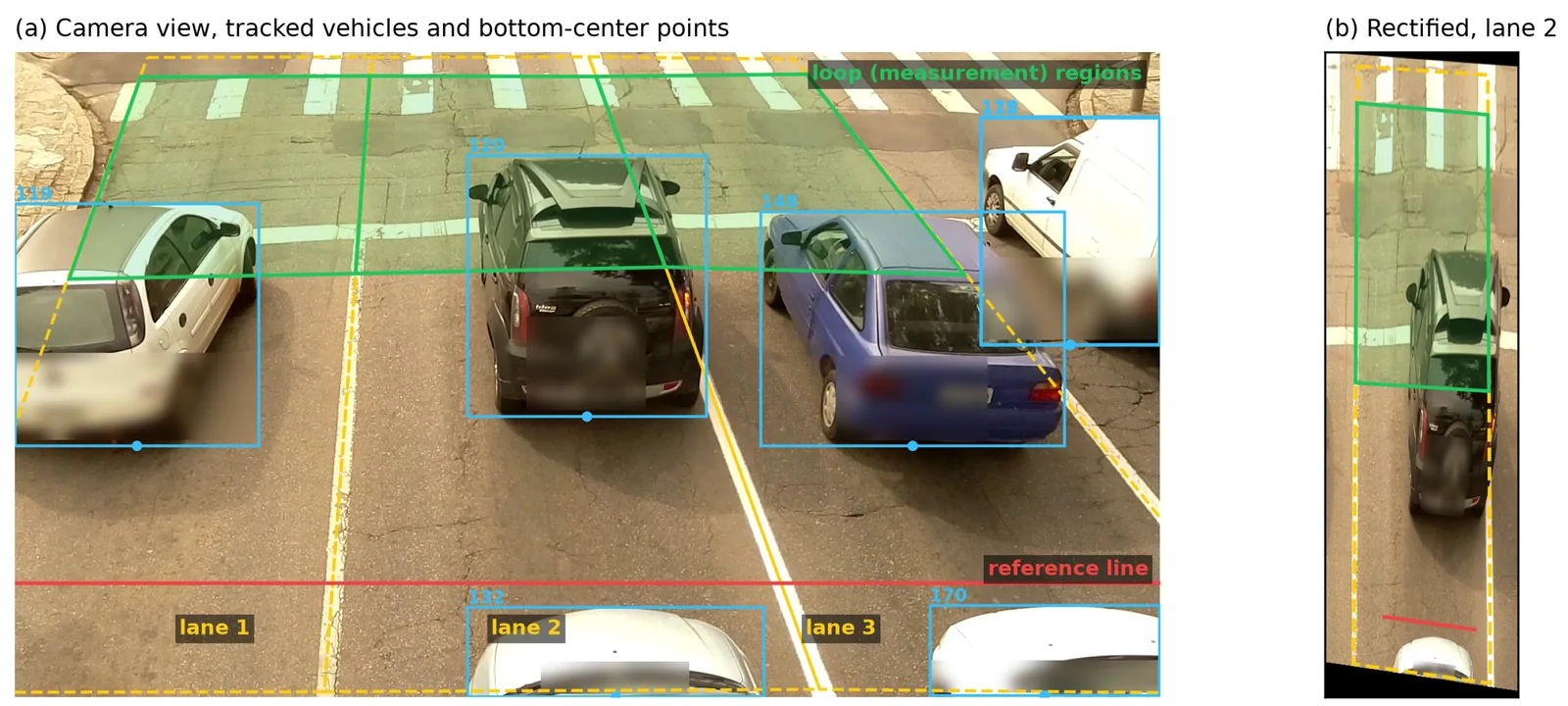
Scene geometry of the evaluation camera. (**a**) Lane regions (dashed yellow), inductive-loop measurement regions (green), the reference line used for headway measurement (red), and tracked vehicles with their identities and bottom-centre reference points (blue) on one frame; licence plates are blurred. (**b**) The middle lane mapped onto the rectified road plane with its homography; the loop region becomes a rectangle and vehicle motion becomes a straight path along the road.

σ is the only free parameter of the geometric model. It is estimated once, on a single calibration video, as the median ratio between the loop-meter speed of each matched vehicle and its speed in rectified pixels per second, and is then kept fixed for all other videos. Because the loop meter measures speed along the road, this calibrates exactly the direction in which speed, headway and spacing are measured. We obtain σ=0.0172 m/px. As a consistency check against a road feature, the nominal 4.8 m length of the loop rectangles drawn in the dataset annotation implies 0.0117–0.0125 m/px in the three lanes; the two differ by a factor of about 1.4, and we discuss this discrepancy in Section 5. The scene geometry is illustrated in Figure 2.

### 3.3. Vehicle Speed

The speed of vehicle *i* at time *t* is the slope $\hat{b}$ of the least-squares line fitted to $s_{i}$ over a centred window of 0.7 s,

(7) $$\left(\hat{a},\hat{b}\right)=\arg\min_{a,b}\sum_{|\tau -t|\leq 0.35\;s}{\left(s_{i}\left(\tau \right)-a-b\;\tau \right)}^{2},\;v_{i}\left(t\right)=\hat{b},$$

which is less sensitive to box jitter than a two-point difference. Time is computed as frame index divided by the frame rate. The speed reported for a vehicle is the median of $v_{i}\left(t\right)$ over the samples in which its contact point lies inside the lane’s measurement region, the road section covered by the reference speed meter.

### 3.4. Time Headway, Spacing and Time to Collision

Headways are measured at a reference line across the road, the image row at which the dataset’s licence plates were annotated. The crossing time $t_{i}$ of each track is obtained by linear interpolation between the two frames on either side of the line. Close to the camera, fast vehicles are often first detected after they have passed the line; for such tracks, the crossing time is extrapolated back from their first six samples, with time modelled as a linear function of image row, when the first detection lies at most 200 px past the line. Vehicles in the same lane are ordered by crossing time, and each vehicle forms a pair with the vehicle that crossed immediately before it, which is its leader. In the evaluated scene, traffic moves away from the camera, so the leader is the vehicle higher in the image.

The time headway of follower *f* behind leader *l* is

(8) $${\mathrm{THW}}_{f}=t_{f}-t_{l},$$

and pairs with $0<{\mathrm{THW}}_{f}\leq 10$ s are retained. When the follower reaches the reference line the leader has usually left the field of view, so the spacing is computed from the headway and the leader’s speed $v_{l}$ (Section 3.3):

(9) $$d_{f}={\mathrm{THW}}_{f}\;v_{l}.$$

**Figure 3:**
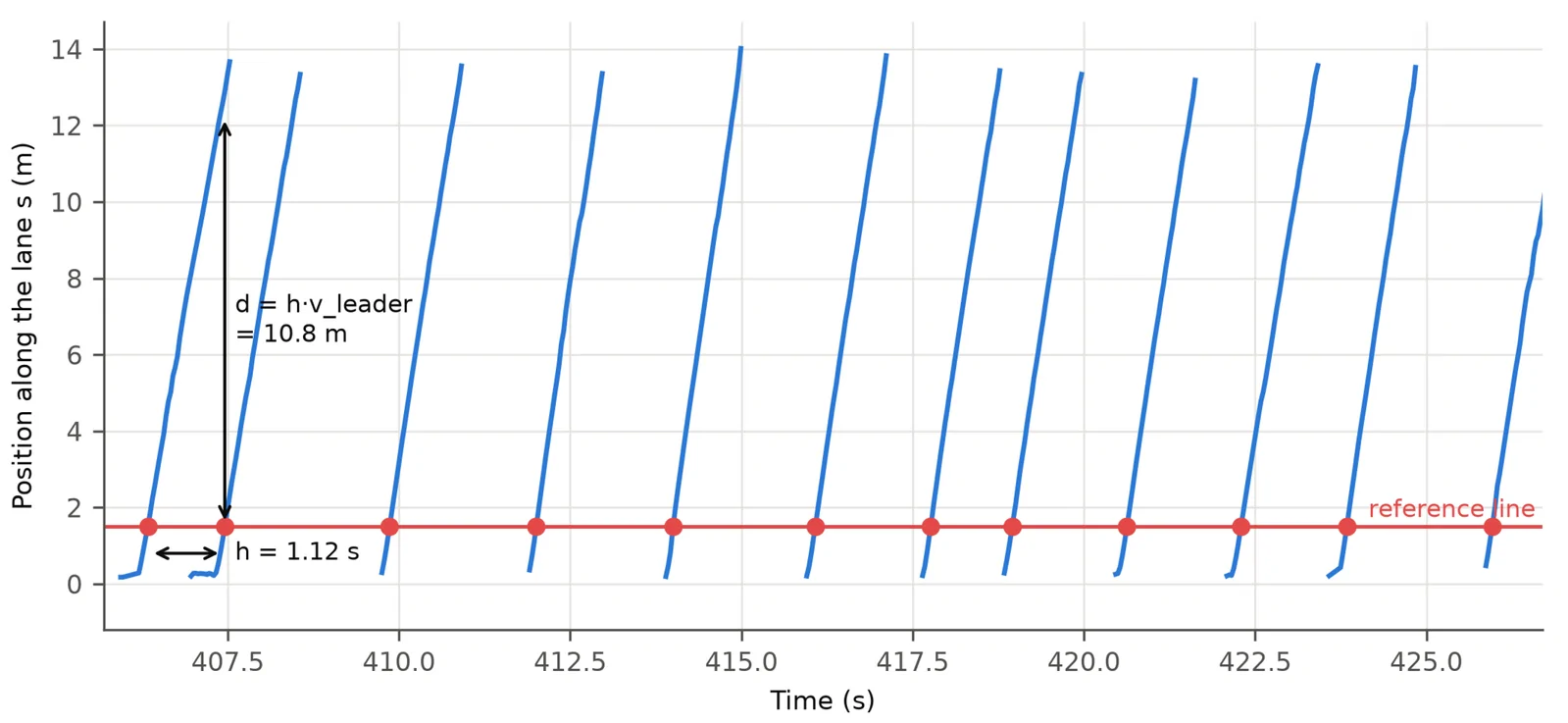
Measured road-coordinate trajectories of 12 consecutive vehicles in one lane. The crossings of the reference line define the time headway *h* of each pair, and the spacing *d* follows from the headway and the leader’s speed.

Because both vehicles are represented by the same reference point at the same line, $d_{f}$ is a front-to-front spacing. The bumper-to-bumper gap is smaller by the length of the leader, which a single camera cannot measure reliably; using $d_{f}$ in a rule defined on the gap therefore errs on the side of not flagging a vehicle. Figure 3 illustrates these quantities on measured trajectories.

When the follower is faster than the leader by more than 0.1 m/s, the time to collision is

(10) $${\mathrm{TTC}}_{f}=\frac{d_{f}}{v_{f}-v_{l}},$$

and it is undefined otherwise.

### 3.5. Violation Criteria

Legal and guideline definitions of a safe following distance differ between countries, so we evaluate several criteria (Table 2). The rule that the distance in metres must not be smaller than half the speed in km/h,

(11) $$d_{f}<\frac{v_{f}^{\mathrm{km}/h}}{2}=\frac{3.6\;v_{f}}{2}=1.8\;s\cdot v_{f},$$

is, based on Equation (9), equivalent to ${\mathrm{THW}}_{f}<1.8\;s\cdot v_{f}/v_{l}$, that is, to a time headway below 1.8 s when the two vehicles travel at the same speed. In Türkiye, Article 101 of the Highway Traffic Regulation [26] requires drivers to keep a safe distance from the vehicle ahead and, for vehicles travelling in convoys or groups, sets this distance to at least half of the maximum speed in metres; the half-speed rule is the common operational form of this requirement. The THW threshold is additionally swept from 1.0 to 2.5 s in steps of 0.1 s.

### 3.6. Uncertainty of the Violation Decision

A violation decision near the threshold is sensitive to small measurement errors. The THW errors of the 89 matched pairs of the calibration video form an empirical error distribution E. Like the scale σ, it is estimated once per camera, so no ground truth of the test videos is used. For each estimated pair, we draw 500 samples e∼E and compute the probability

(12) $$p_{f}=\mathrm{Pr}\left({\mathrm{THW}}_{f}-e<1.8\;s\right)$$

that the true headway is below 1.8 s. Pairs with $0.1<p_{f}<0.9$ are labelled *uncertain*: an enforcement system should not decide them automatically. Followers with $p_{f}\geq 0.9$ are flagged automatically and followers with $p_{f}\leq 0.1$ are cleared automatically. To test how much the result depends on the source of E, the distribution was also estimated leave-one-video-out, from the matched pairs of all other videos.

The main parameters are summarized in Table 3.

## 4. Results

The measurements were validated on the public UTFPR urban vehicle-speed dataset [8]. A fixed camera records a three-lane urban road at 1920×1080 pixels and 30.15 frames per second. For every vehicle, the dataset provides the lane, the frame and bounding box of its first readable licence plate and, for most vehicles, the speed measured by an inductive-loop speed meter. The videos are grouped into subsets by recording condition (Table 4). We processed 15 videos (182 min, 6058 vehicles, 5454 of them with a loop speed, 277 motorcycles). Video 1 of subset 01 was used only to estimate the along-road scale σ; all results below are reported on the remaining 14 test videos (178 min, 5939 vehicles). In video 2 of subset 04, only the first 40,600 of 54,380 frames are annotated, and the unannotated remainder was excluded from the evaluation.

Three reference values were used. The reference speed is the loop-meter speed, which is an independent measurement. The reference time headway is the difference between the plate-annotation frames of two consecutive vehicles in the same lane divided by the frame rate; it is derived from manual annotation with a resolution of one frame (33 ms). The reference spacing is the reference headway multiplied by the leader’s loop speed; it assumes a constant leader speed over one headway and does not depend on σ. An estimated vehicle was matched to a reference vehicle in the same lane when its crossing of the plate row was at most three frames from the annotated plate frame. Speed, headway and spacing errors are reported as mean error, mean absolute error (MAE), root-mean-square error (RMSE) and 95th percentile of the absolute error (P95); for speed, we also report the share of vehicles inside the −3/+2 km/h tolerance band used by Luvizon et al. [8]. Violation detection is evaluated per follower vehicle with precision, recall and F1, where a reference violation is obtained by applying the same criterion to the reference values. Coverage is reported as vehicle recall (matched reference vehicles) and pair recall (matched reference headway pairs). All values are pooled over the matched vehicles or pairs of the 14 test videos. Runtime is the detection and tracking time per frame on an NVIDIA RTX 3060 Ti (8 GB) with an AMD Ryzen 5 5600X processor, using PyTorch 2.11, CUDA 12.8, Ultralytics 8.4 and BoxMOT 25.0.

**Figure 4:**
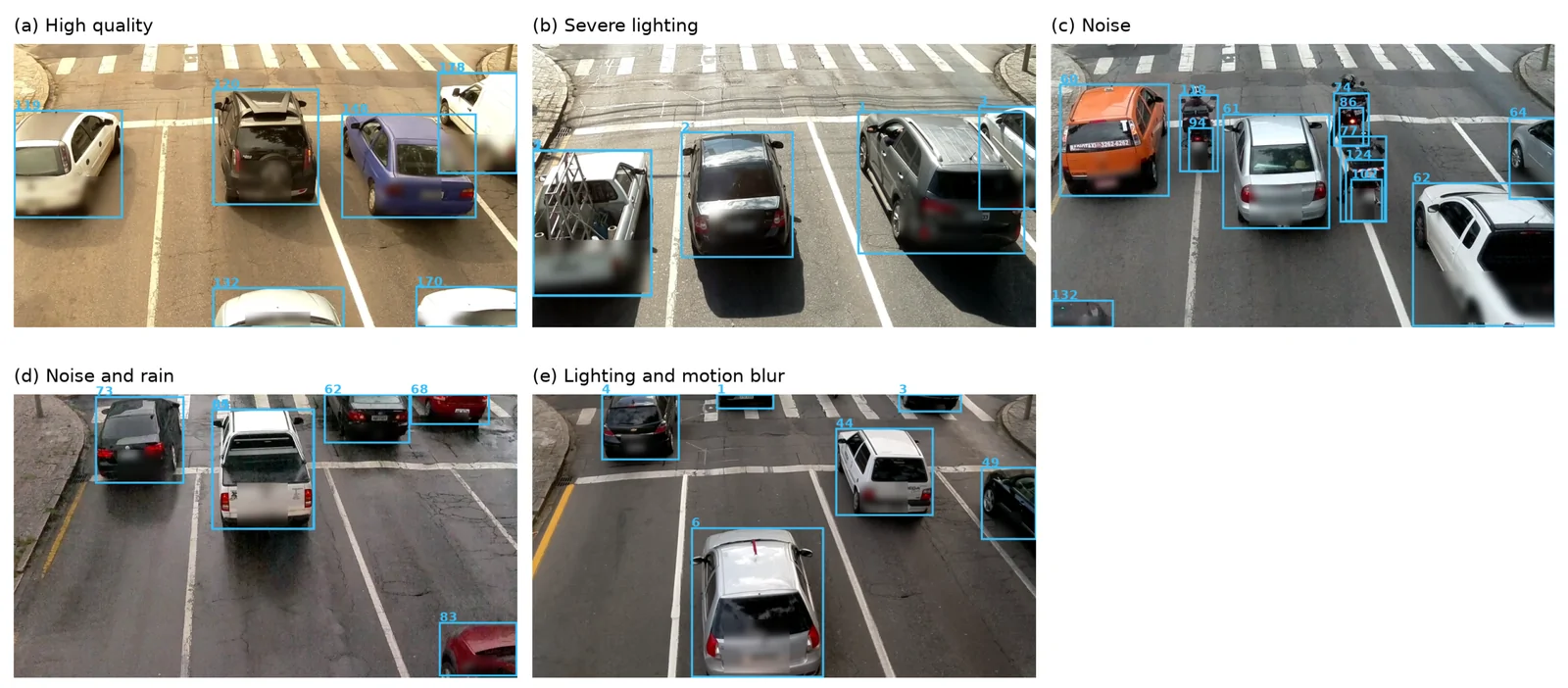
Example frames of the five UTFPR recording conditions with the tracked boxes and identities of YOLO11-m + BoT-SORT. Licence plates are blurred.

Figure 4 shows the tracker output on one frame of each recording condition.

**Figure 5:**
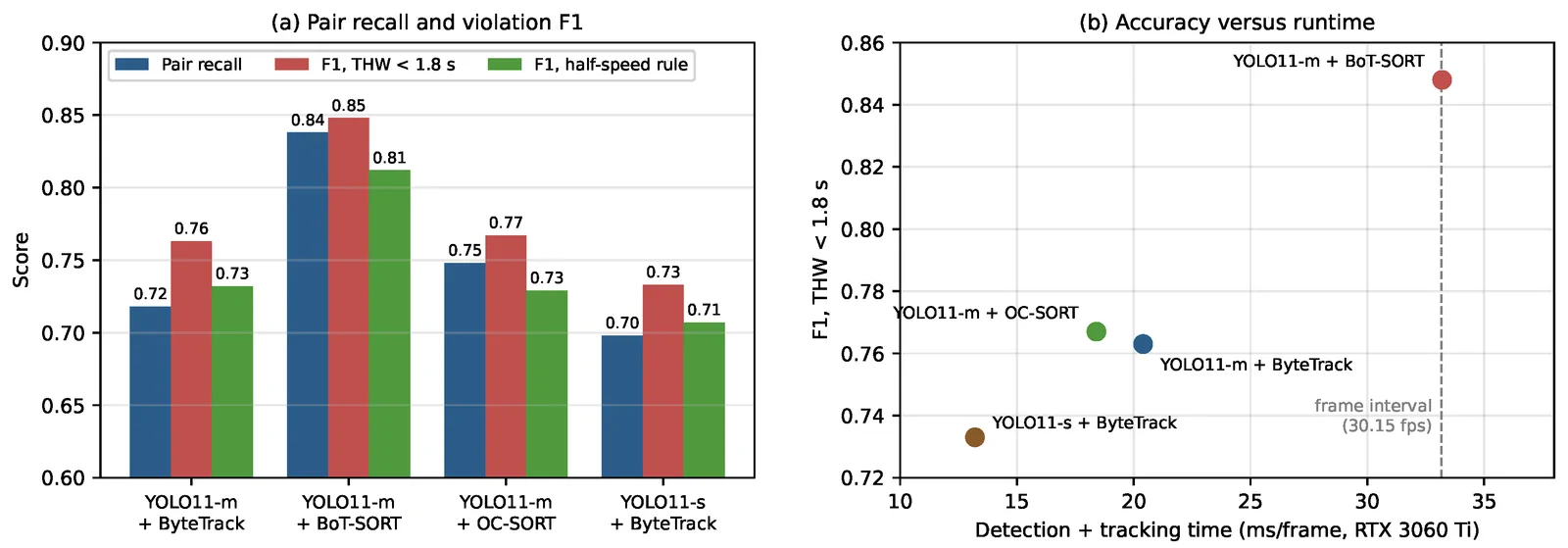
Comparison of the four configurations on the 14 UTFPR test videos. (**a**) Headway-pair recall and violation F1. (**b**) F1 of the 1.8 s headway criterion against detection and tracking time per frame; the dashed line marks the frame interval of the videos.

Table 5 and Figure 5 compare the four detector and tracker configurations. All of them find 94–96% of the reference vehicles, and all of them measure speed and headway with similar accuracy: the speed MAE lies between 1.04 and 1.19 km/h, 86–91% of the speeds fall inside the tolerance band, and the headway MAE lies between 0.057 and 0.060 s. The configurations differ mainly in how many headway pairs they recover, which depends on keeping both vehicles of a pair under one identity until the reference line. BoT-SORT recovers 83.8% of the reference pairs, against 69.8–74.8% for the other configurations, and therefore also has the lowest spacing error (1.14 m) and the highest violation F1 (0.848 for THW<1.8 s and 0.812 for the half-speed rule). Its cost is runtime: at 33.2 ms per frame, it runs at about the 33 ms frame interval of the videos, whereas YOLO11-s with ByteTrack needs 13.2 ms per frame at a moderate loss of pair recall and F1.

**Figure 6:**
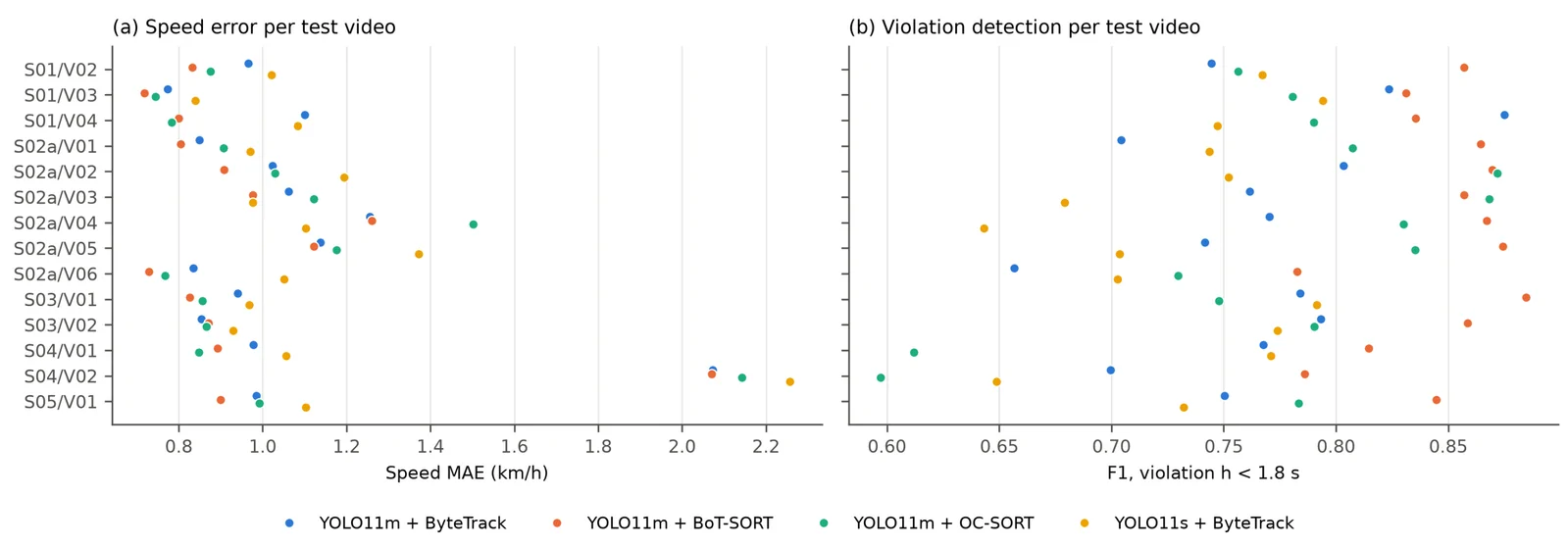
Speed MAE and F1 of the 1.8 s headway criterion of the four configurations in each of the 14 UTFPR test videos.

The ranking of the configurations is consistent across videos (Figure 6): BoT-SORT gives the highest F1 of the 1.8 s criterion in 11 of the 14 test videos.

**Figure 7:**
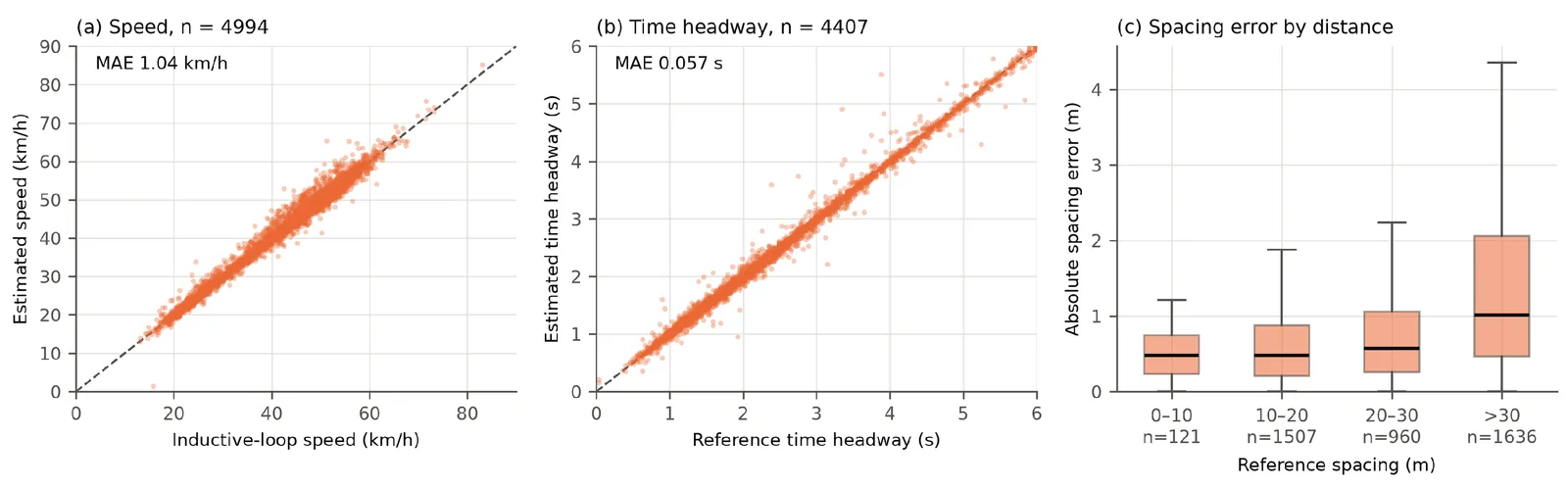
Measurement accuracy of YOLO11-m + BoT-SORT on the 14 UTFPR test videos. (**a**) Estimated speed against the inductive-loop speed. (**b**) Estimated against reference time headway (pairs up to 6 s shown). (**c**) Absolute spacing error grouped by reference spacing; boxes show the interquartile range and the median, whiskers 1.5 times the interquartile range.

Table 6 and Figure 7 show the errors of the best configuration, YOLO11-m with BoT-SORT. The estimated speeds follow the loop-meter speeds over the whole range from about 10 to 80 km/h. The mean speed error is +0.12 km/h, so the scale σ estimated on the calibration video carries over to the other videos without a noticeable bias, and the speed MAE is similar in the three lanes (1.01, 0.90 and 1.18 km/h). The estimated headways lie close to the reference values, with an MAE of 0.057 s, less than two frames. The spacing error grows with the spacing itself, from an MAE of 0.65 m below 10 m to 0.68 m between 10 and 20 m, 0.90 m between 20 and 30 m and 1.73 m above 30 m, as expected from $d_{f}={\mathrm{THW}}_{f}\;v_{l}$, in which a fixed relative speed error produces an absolute spacing error proportional to the spacing. At the short spacings that matter for a violation decision, the error is therefore below 1 m. Relative to the reference values, the mean absolute percentage error is 2.5% for speed, 2.7% for time headway and 3.9% for spacing. None of the three quantities is noticeably biased: the mean error is +0.004 s (±0.004 s, 95% confidence interval) for the headway, −0.001 s for reference headways between 1.3 and 2.3 s, and +0.07 m (±0.06 m) for the spacing.

**Figure 8:**
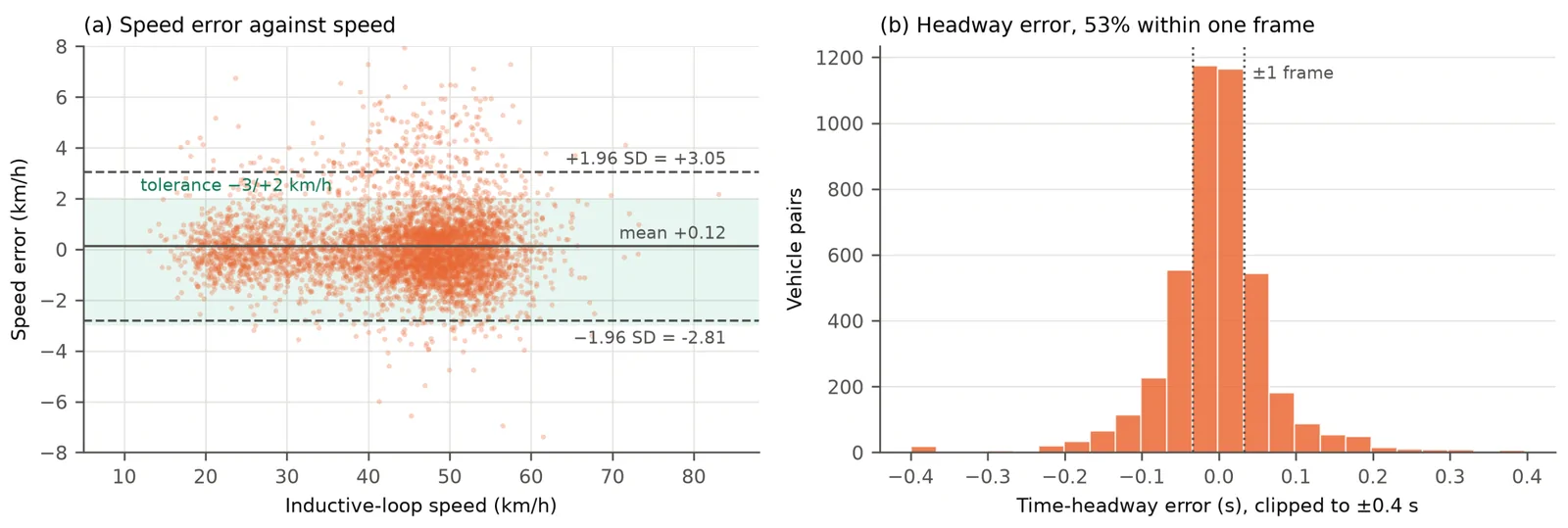
Error distributions of YOLO11-m + BoT-SORT on the 14 UTFPR test videos. (**a**) Speed error against loop speed with the mean and the 95% limits of agreement. (**b**) Histogram of the time-headway error.

Figure 8 examines the error distributions in more detail. The speed error shows no trend with speed, and its 95% limits of agreement are −2.8 and +3.1 km/h. Of the headway errors, 53% are smaller than one frame and 87% smaller than three frames.

**Figure 9:**
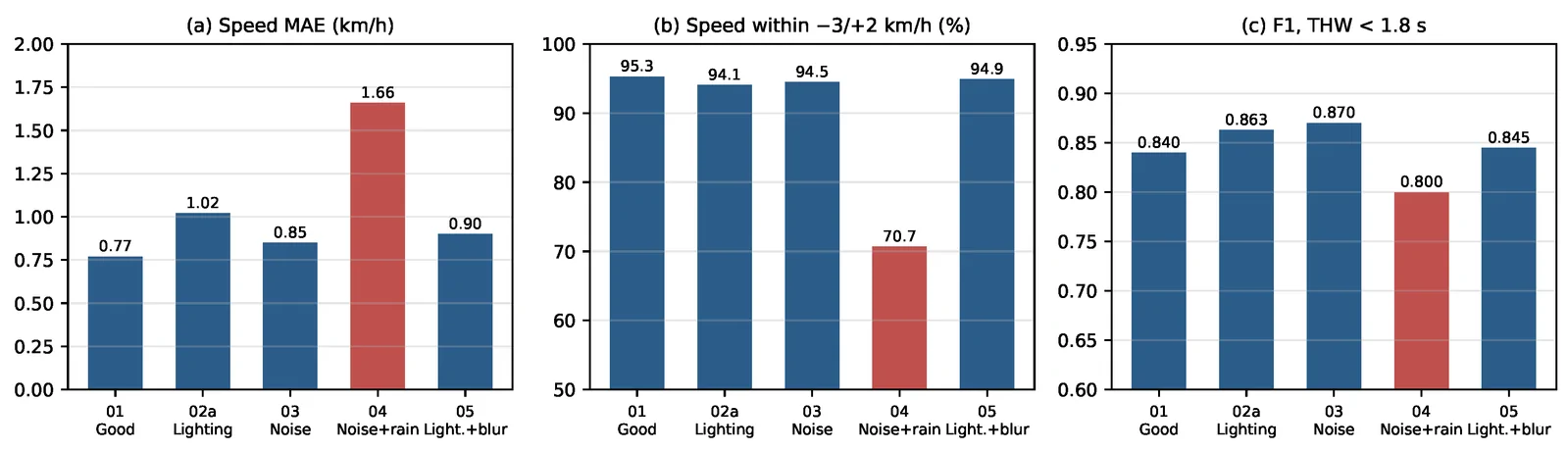
Results of YOLO11-m + BoT-SORT by recording condition. (**a**) Speed MAE. (**b**) Share of speeds within the −3/+2 km/h tolerance. (**c**) F1 of the 1.8 s headway criterion. The noise and rain subset is highlighted.

The effect of the recording condition is shown in Table 7 and Figure 9. Strong lighting changes, image noise and motion blur change the results only slightly: the speed MAE stays between 0.77 and 1.02 km/h and 94–95% of the speeds fall inside the tolerance band. The noise and rain subset is the most difficult: its speed MAE rises to 1.66 km/h, only 70.7% of the speeds remain inside the band, and the F1 of the 1.8 s criterion drops to 0.800. Headway is the most robust quantity, with an MAE between 0.051 and 0.065 s in all conditions, because it depends only on the time at which a vehicle crosses the reference line and not on the metric scale.

Table 8 reports the violation criteria of Table 2 for YOLO11-m with BoT-SORT. The time-headway criteria are detected most reliably: for THW<1.8 s, the system finds 1691 of the 1855 reference violations (recall 0.912) with a precision of 0.793, and for THW<2.0 s the F1 reaches 0.866. The half-speed rule reaches a similar recall (0.928) but a lower precision (0.722), because it combines the measured headway with the ratio of two measured speeds, whereas the headway criterion depends on the headway alone. For all criteria, false positives outnumber missed violations. This is not caused by a bias of the measured headway (Table 6). For the 1.8 s criterion, measurement errors close to the threshold act in both directions about equally often: 56 false positives have a reference headway between 1.8 and 2.2 s, and 45 missed violations were measured between 1.8 and 2.2 s. Most false positives come from association errors instead. In 274 of the 442 cases, the flagged follower could not be matched to any annotated vehicle, for example, a fragmented track or a duplicate detection that forms a spurious pair with a short headway; in 22 cases, the matched follower has no reference pair, and in a further 90 cases, the reference headway is at least 2.2 s, which probably means that a spurious track was taken as the leader. Of the 164 missed violations, 95 have no estimated pair because one of the two vehicles was not tracked across the reference line. The fixed 10 m criterion performs poorly (precision 0.222, F1 0.344): because the spacing is measured front to front, a spacing below 10 m leaves only a few metres between the vehicles, and such pairs are rare (152 reference violations), so a small number of false positives dominates the precision. The fixed 20 m criterion, with more positives, reaches an F1 of 0.802. The time-to-collision criteria cannot be assessed: only 4 and 34 followers in almost three hours of urban traffic have a reference TTC below 3 s and 4 s, so their scores are not meaningful.

**Figure 10:**
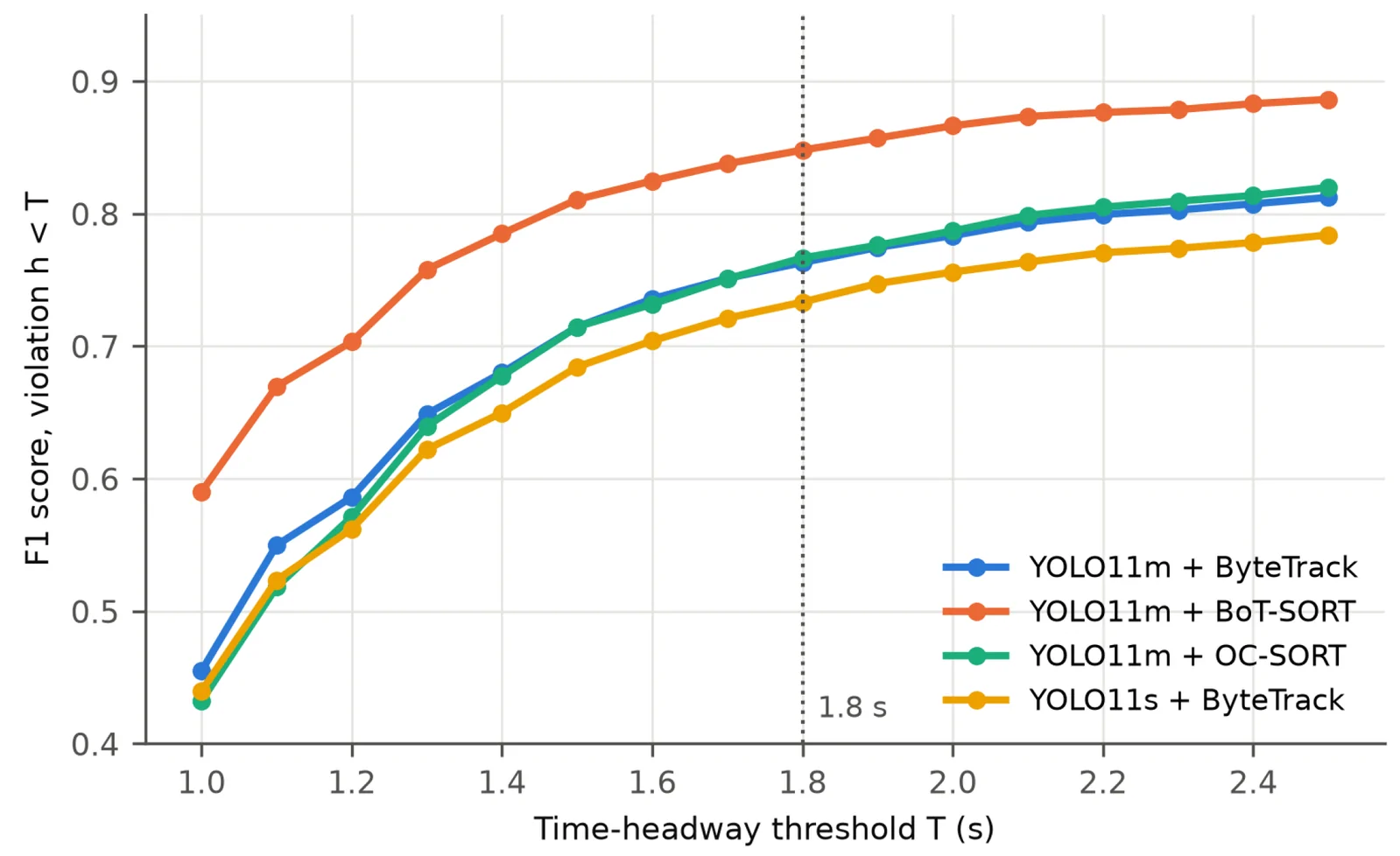
F1 of time-headway violation detection as a function of the threshold for the four configurations (14 UTFPR test videos).

Figure 10 shows how the F1 of the time-headway criterion changes with its threshold. For all configurations, F1 increases with the threshold. Because the headway error is nearly constant, the rise mainly reflects the number of positives: short headways are rare, and a small timing error changes the decision for a larger share of them. At 1.0 s, the F1 of BoT-SORT is 0.59 and that of the other configurations 0.43–0.46; above 1.5 s, BoT-SORT stays above 0.81 and the other configurations lie between 0.68 and 0.82. BoT-SORT has the highest F1 at every threshold. Thresholds in the range used by traffic codes, around 1.8–2.0 s, are therefore measured more reliably than a strict 1.0 s threshold.

**Figure 11:**
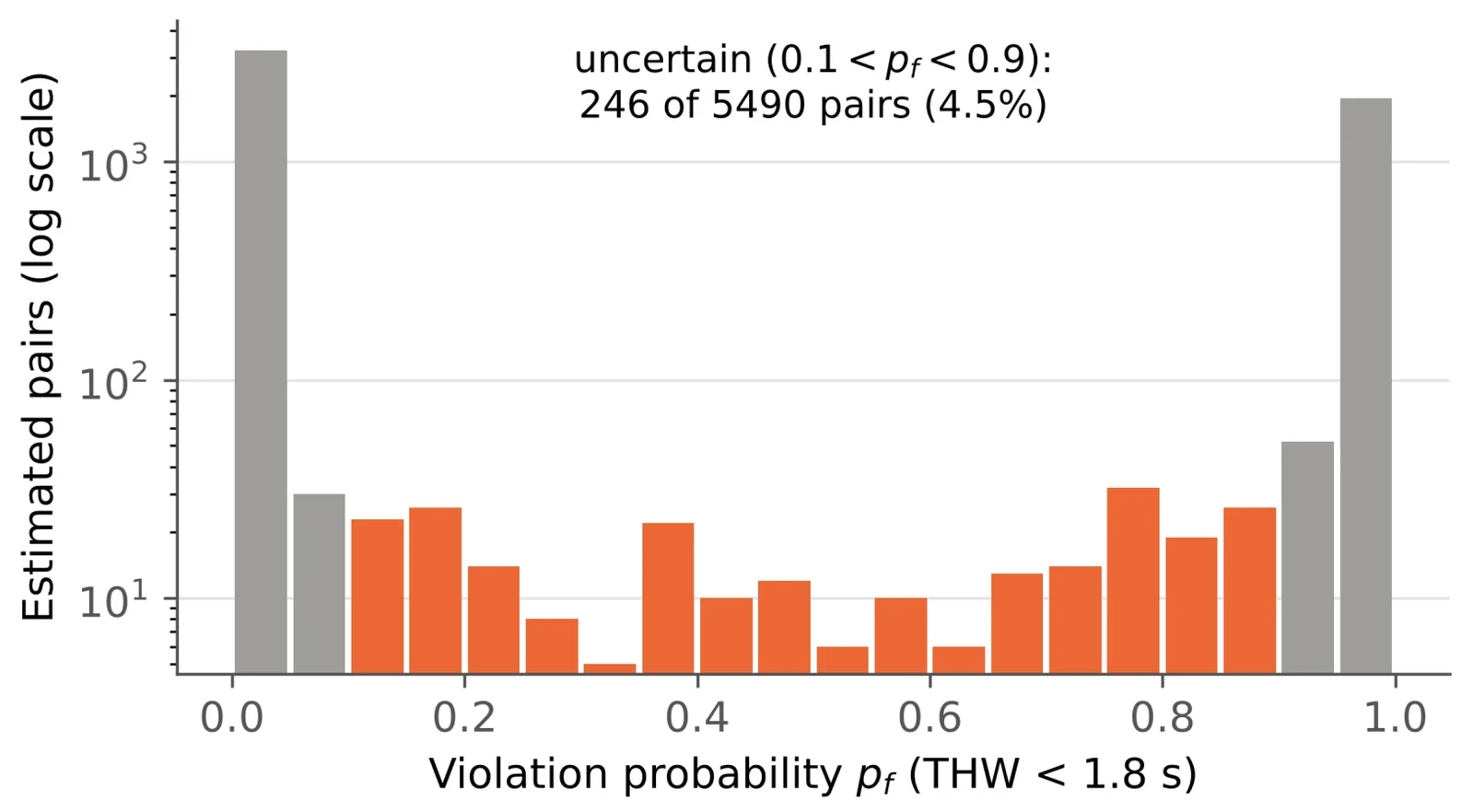
Distribution of the violation probability $p_{f}$ of the 5490 estimated pairs for the 1.8 s criterion (YOLO11-m + BoT-SORT). The error distribution is estimated on the calibration video. Pairs with $0.1<p_{f}<0.9$ (4.5%) are labelled uncertain.

With the error distribution estimated on the calibration video, 246 of the 5490 estimated pairs (4.5%) have a violation probability between 0.1 and 0.9 for the 1.8 s criterion (Figure 11). Estimated leave-one-video-out, the distribution gives 266 uncertain pairs (4.8%), and taken from the matched pairs of each test video itself, which would not be possible in deployment, 267 (4.9%). The share of uncertain pairs therefore hardly depends on the ground truth of the test videos (4.5–4.9%). Table 9 reports the automated decisions. If only followers with $p_{f}\geq 0.9$ are flagged automatically, 1996 followers are flagged with a precision of 0.797 and a recall of 0.858, against 0.793 and 0.912 for the hard threshold; 246 followers, 130 of them with a reference violation, are routed to an operator, and 39 reference violations are cleared automatically. The automated decisions are hardly more precise than the hard threshold because $p_{f}$ models only the headway measurement error, which is small, whereas most false positives are caused by association errors that this error model does not capture. Among the automatically flagged followers that could be matched to an annotated vehicle, the precision is 0.920 (1730 followers). For enforcement, the precision of automated decisions is therefore limited by tracking and association rather than by the accuracy of the headway measurement.

UTFPR has no per-frame bounding-box annotations, so detection and tracking accuracy were measured separately on the 40 test sequences of UA-DETRAC [9] (960×540 pixels, 25 frames per second; 56,167 annotated frames, 675,774 boxes, 2337 vehicle identities), using the CC-BY-4.0 copy of the dataset published on Hugging Face. The detectors were used with the same COCO weights and settings as on UTFPR and evaluated class-agnostically; detections lying more than half inside an annotated ignored region were discarded. Average precision (AP) was computed with 101-point interpolation at an intersection-over-union threshold of 0.5 (AP@0.5) and averaged over thresholds from 0.5 to 0.95 (AP@0.5:0.95). Tracking was evaluated with the CLEAR MOT metrics [15], IDF1 [16] and HOTA [17]; our implementation of these metrics was checked against the TrackEval and pycocotools reference implementations. The two detectors perform almost identically (Table 10): YOLO11-m reaches an AP@0.5 of 0.837 and an AP@0.5:0.95 of 0.622, and YOLO11-s 0.842 and 0.617 with less than half the parameters and one third of the computation.

The tracking results (Table 11) rank the trackers in the same order as the measurement results on UTFPR. BoT-SORT has the highest HOTA (0.681), MOTA (0.684) and IDF1 (0.790) and the fewest identity switches (662, against 998 for ByteTrack and 1853 for OC-SORT). Its advantage lies mainly in association (AssA 0.745), which is the property that preserves headway pairs on UTFPR; identity preservation is therefore a suitable criterion for choosing a tracker for following-distance analysis.

**Figure 12:**
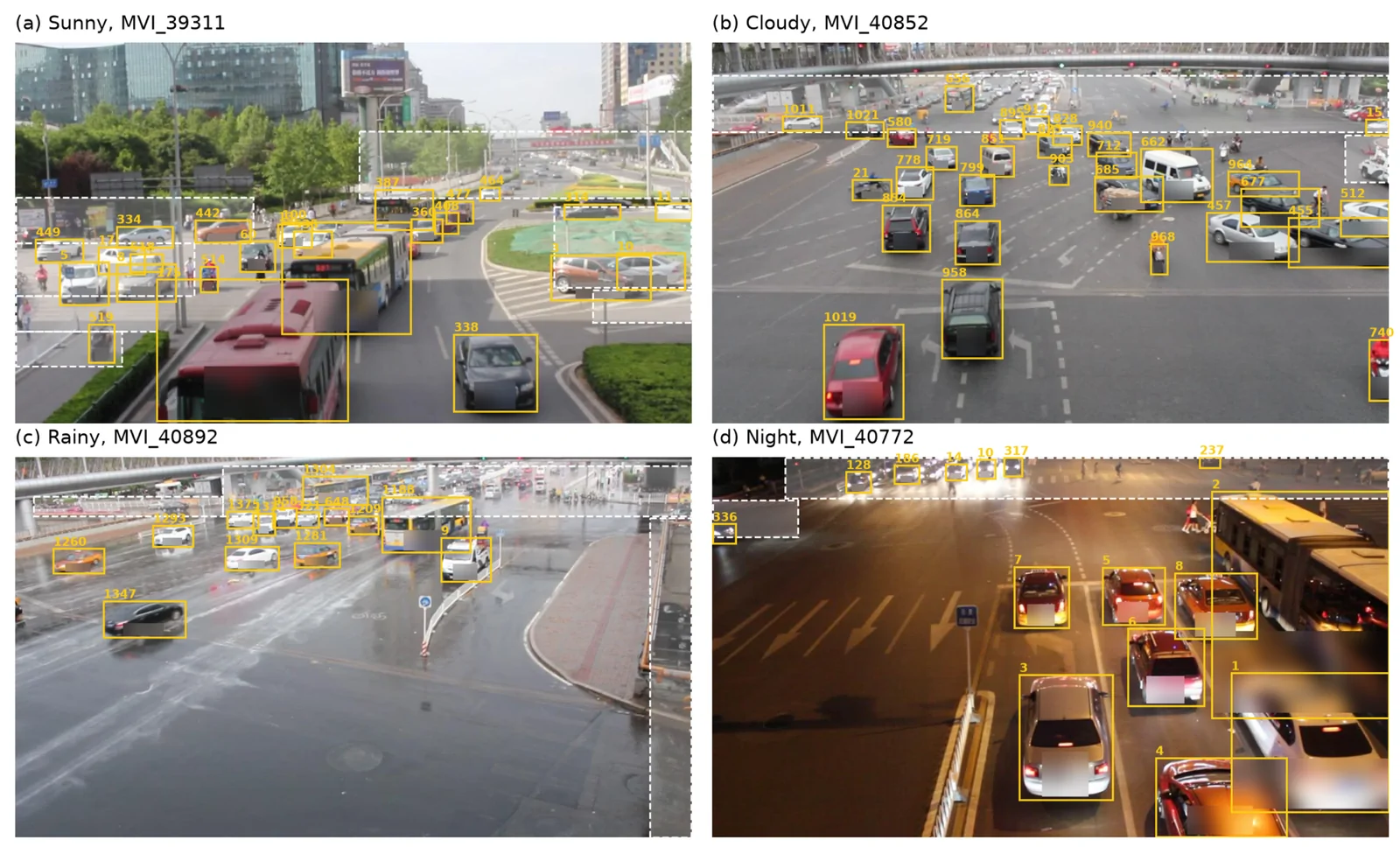
Example UA-DETRAC test frames in the four weather conditions (sunny, cloudy, rainy, night) with the annotated ignored regions.

**Figure 13:**
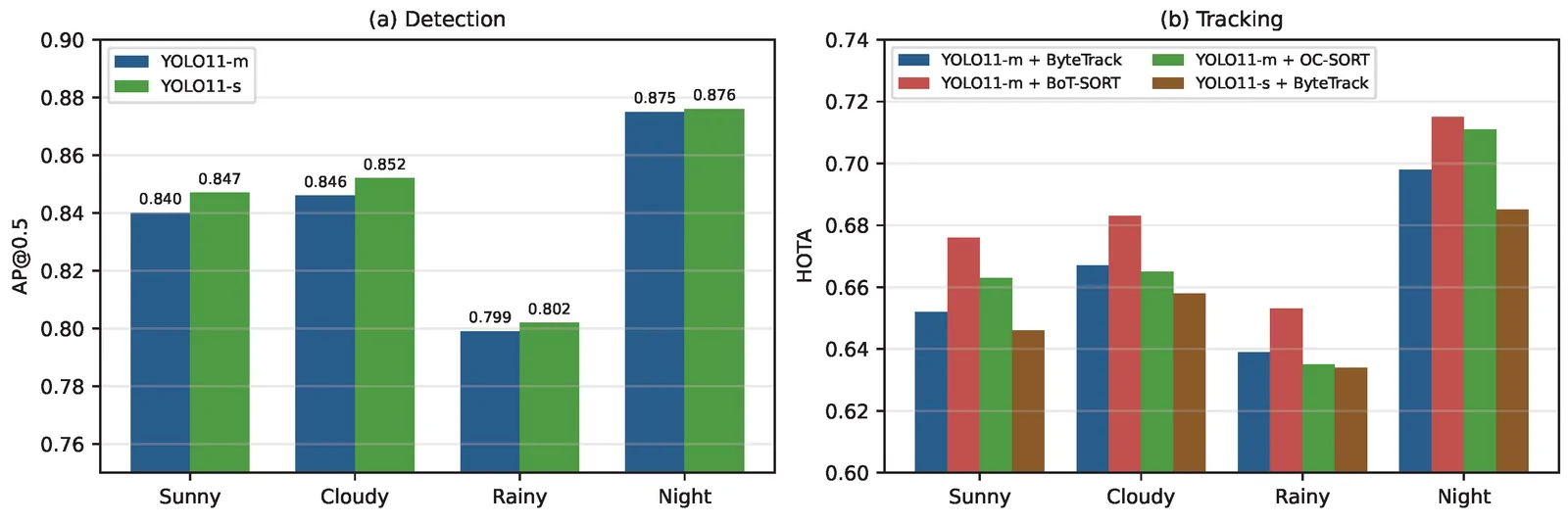
Detection and tracking on UA-DETRAC by weather condition. (**a**) AP@0.5 of the two detectors. (**b**) HOTA of the four configurations.

The copy of UA-DETRAC used here carries no weather attribute, so each test sequence was assigned to one of the benchmark’s four conditions by visual inspection of its frames: sunny (hard shadows, 10 sequences), cloudy (diffuse light and dry road, 9), rainy (wet road and reflections, 9) and night (street lighting, 12, two of them at dusk). Example frames of the four conditions are shown in Figure 12. Table 12 and Figure 13 show that rain is the most difficult condition for both detection (AP@0.5 0.799 for YOLO11-m, against 0.840–0.875 in the other conditions) and association (lowest HOTA and IDF1 and most identity switches for every tracker), in agreement with the noise and rain subset of UTFPR. BoT-SORT has the highest HOTA and IDF1 in every condition. The night sequences obtain the highest scores, but they come from a few well-lit and comparatively sparse junctions, whereas the cloudy and rainy groups contain the densest scenes of the test set; this result therefore reflects the scenes of this test set rather than an advantage of night-time operation.

## 5. Discussion

The results show that a fixed monocular surveillance camera can measure the quantities on which following-distance rules are based with an accuracy that is known and adequate for screening. With the best configuration, speeds were measured with an MAE of 1.04 km/h against an inductive-loop meter, time headways with an MAE of 0.057 s, less than two video frames, and spacings with an MAE of 1.14 m, which falls below 1 m for the short spacings that matter for a violation. The contribution of this study therefore does not lie in a new detector or tracker but in a measurement chain whose every output, from speed to the final violation decision, has been compared with reference values. This distinguishes the framework from systems that demonstrate the detection of unsafe following only visually, and it supports recent work that uses headways from ordinary traffic cameras as crash-related indicators [4], because such indicators are only as reliable as the underlying measurement.

The most consequential design decision was the metric scale. Perspective correction with a homography makes distances along the road comparable across the image [5], but it does not fix their absolute size, and the along-road scale is the main source of error in camera-based speed measurement [6]. In this study, the scale obtained from loop speeds, σ=0.0172 m/px, differs from the 0.0117–0.0125 m/px implied in the three lanes by the nominal 4.8 m length of the loop rectangles drawn in the dataset annotation. With the rectangle-based scale, all estimated speeds and spacings would be about 30% too small, which is incompatible with the loop measurements on all 14 test videos. Since speeds are measured inside these rectangles, the discrepancy indicates either that the annotated rectangles do not correspond to 4.8 m of road in the rectified view or that the supplied homographies are not metrically consistent along the road; we could not determine which from the available data. In either case, a scale taken from nominal road dimensions, such as marking lengths or lane widths, would have produced a large systematic error, whereas calibration against a small number of reference speeds removed it: a single 3.8-min video with 119 vehicles was sufficient, and the resulting scale carried over to the other 14 videos with a mean speed error of +0.12 km/h. For deployment, this suggests calibrating each camera against a few independently measured speeds, for example from a loop detector or a probe vehicle with satellite positioning, rather than relying on road geometry alone. Automatic calibration methods for traffic cameras [18,19,20] could reduce this effort, but their accuracy would need to be verified in the same way. Because the same type of sensor serves for calibration and validation here, a systematic bias of the loop meter would propagate to our speeds; the reference headways, which come from frame counts, are not affected by it.

The comparison of configurations shows that the tracker matters more than the detector for following-distance analysis. YOLO11-s and YOLO11-m reached almost the same detection accuracy on UA-DETRAC, and all configurations measured individual speeds and headways with similar error. What differed was the share of headway pairs recovered, which requires both vehicles of a pair to keep their identities until they cross the reference line. BoT-SORT recovered 83.8% of the pairs, against 69.8–74.8% for ByteTrack and OC-SORT, and the same ranking appeared in the independent UA-DETRAC evaluation, where BoT-SORT had the highest association accuracy and the fewest identity switches in every weather condition. Association-oriented tracking metrics such as AssA and IDF1 [16,17] are therefore a better guide for choosing a tracker for this task than detection accuracy alone. The price of BoT-SORT is runtime: at 33.2 ms per frame on a mid-range GPU it runs at the frame interval of the video, whereas YOLO11-s with ByteTrack needs 13.2 ms per frame at a loss of about 0.11 in F1 for the 1.8 s criterion. The choice between them depends on whether one camera or several cameras share a processor.

The violation criteria differ considerably in how reliably they can be measured. Time-headway criteria were the most robust, because the headway depends only on the times at which two vehicles cross a line and not on the metric scale: its error changed little between recording conditions, even in the rain and noise subset where the speed error increased. The half-speed rule, which equals a 1.8 s headway when both vehicles travel at the same speed, reached a slightly lower F1 (0.81 against 0.85) because it also depends on the ratio of two measured speeds. A fixed 10 m distance was unreliable, mainly because front-to-front spacings below 10 m are rare. Time to collision, although an established surrogate safety indicator [3,24,25], could not be evaluated: closing-speed conflicts with a TTC below 4 s occurred only 34 times in almost three hours of urban traffic. Evaluating TTC-based rules will require data with more interactions, such as dense or congested traffic or conflict-oriented recordings [21,23]. From the measurement point of view, headway-based rules are therefore the most suitable basis for automated enforcement, which is consistent with the traffic codes that state the following distance as a function of speed.

The remaining decision errors are mostly false positives: for the 1.8 s criterion, 442 followers were flagged without a reference violation, against 164 missed violations. Two properties of the framework limit their impact. First, for the distance-based criteria, the spacing is measured front to front and is therefore larger than the bumper-to-bumper gap, so a rule defined on the gap is applied conservatively. Second, the probabilistic formulation identifies the pairs whose headway is too close to the threshold for a reliable decision: 4.5% of the pairs were labelled uncertain with an error model taken from the calibration video alone. The automated decisions ($p_{f}\geq 0.9$) nevertheless reached a precision of only 0.80, about the same as the hard threshold, because the headway measurement error is small and almost unbiased, whereas most false positives arise from association errors, which a measurement-error model cannot detect. An operational system should therefore combine the probability with checks on the tracks themselves, such as track continuity through the measurement region and a plausibility check of the leader, or keep a human confirmation for every flagged follower until such checks have been validated. Detection and tracking were weakest in rain on both datasets, where reflections on the wet road and, in UA-DETRAC, the densest scenes coincide; a deployment should report results per weather condition rather than as a single average.

This study has several limitations. All UTFPR videos come from a single camera site; the recording conditions vary, but the viewpoint and road geometry do not, so the transfer to other viewpoints, lens distortions and road layouts is not tested. The homography assumes a planar road, which may not hold on gradients or curved sections. The reference headway is derived from manual plate annotations, with a resolution of one frame, and the reference spacing from that headway and the loop speed, so neither is an independent distance measurement. The spacing is front to front; a bumper-to-bumper gap would require the length of the leading vehicle, which a single camera cannot measure reliably. The weather conditions of UA-DETRAC were labelled visually, and the high night-time scores reflect the particular night scenes of its test set rather than an advantage of night-time operation. Finally, the time-to-collision criteria could not be evaluated because conflicts of this severity are rare in the recorded traffic.

Future work should therefore evaluate the framework at additional camera sites with independent distance references, for example from instrumented probe vehicles or drone recordings, combine it with automatic camera calibration, estimate vehicle length to obtain the bumper-to-bumper gap, and test time-to-collision criteria in denser traffic. Running the lighter configuration on embedded hardware close to the camera is a further step towards practical deployment.

In conclusion, a fixed surveillance camera, combined with current detection and tracking methods, a per-lane homography and an along-road scale calibrated from measured speeds, can measure vehicle speed, time headway and spacing with errors of about 1 km/h, 0.06 s and 1 m, and it can detect violations of the half-speed rule and of a 1.8 s headway with F1 scores of 0.81 and 0.85. Headway-based criteria are measured more reliably than fixed-distance or time-to-collision criteria, the tracker’s ability to preserve identities determines how many vehicle pairs can be evaluated, and the metric scale must be calibrated on site rather than inferred from road dimensions. Together with an explicit estimate of decision uncertainty, these results provide a validated basis for camera-based following-distance monitoring.

## Figures and Tables

**Table 1:** Stages of the framework.

Stage	Method	Output
Vehicle detection	YOLO11-m/YOLO11-s, COCO weights	Vehicle bounding boxes in the original image
Tracking	ByteTrack, BoT-SORT or OC-SORT	Vehicle identities and image trajectories
Road mapping	Per-lane homography and along-road scale σ	Longitudinal road coordinate s(t) in metres
Speed	Least-squares slope of s(t)	Vehicle speed in the measurement region
Headway	Crossing times of a reference line	Time headway and spacing of each pair
Violation analysis	Eight criteria (Table 2)	Violation decision and its uncertainty

**Table 3:** Main parameters of the framework.

Parameter	Value
Detectors	YOLO11-m, YOLO11-s (COCO weights)
Classes	car, motorcycle, bus, truck
Input size/confidence	960 px/0.25 (OC-SORT: 0.1)
Trackers	ByteTrack, BoT-SORT (Ultralytics), OC-SORT (BoxMOT 25.0.0)
Reference point	Bottom-centre of the box (original image)
Gap handling	Split >0.5 s, interpolate ≤0.5 s
Road mapping	Per-lane homography, longitudinal coordinate only
Along-road scale σ	0.0172 m/px, from loop speeds on one calibration video
Speed estimator	Least-squares slope over 0.7 s, median in measurement region
Headway	Crossing of the plate row, interpolated; 0<THW≤10 s
Spacing	$d_{f}={\mathrm{THW}}_{f}\;v_{l}$ (front-to-front)
Uncertainty	500 samples of the THW error on the calibration video; uncertain if 0.1<p<0.9, automatic decision otherwise

**Table 4:** UTFPR videos used in this study.

Subset	Condition	Videos (min)	Vehicles (with Loop Speed)
01	Good quality, daylight	4 (34.1) ^1^	1144 (1031)
02	Strong lighting changes	6 (68.4)	2042 (1825)
03	Image noise	2 (26.1)	958 (876)
04	Noise and rain	2 (33.4)	1045 (927)
05	Lighting changes and motion blur	1 (20.0)	869 (795)

^1^ Including the calibration video.

**Table 5:** Results of the four configurations on the 14 UTFPR test videos (pooled over vehicles and pairs). In tol.: share of speed errors within −3/+2 km/h.

	Recall		Speed		THW	Spacing	F1		Time
Configuration	Vehicles	Pairs	MAE (km/h)	In Tol. (%)	MAE (s)	MAE (m)	THW < 1.8 s	Half-Speed	(ms/Frame)
YOLO11-m + ByteTrack	0.945	0.718	1.10	89.6	0.059	1.37	0.763	0.732	20.4
YOLO11-m + BoT-SORT	**0.958**	**0.838**	**1.04**	**90.6**	**0.057**	**1.14**	**0.848**	**0.812**	33.2
YOLO11-m + OC-SORT	0.938	0.748	1.09	90.5	0.060	1.70	0.767	0.729	18.4
YOLO11-s + ByteTrack	0.940	0.698	1.19	85.9	0.059	1.49	0.733	0.707	**13.2**

**Table 6:** Measurement errors of YOLO11-m + BoT-SORT pooled over the 14 UTFPR test videos.

Quantity	*n*	Mean Error	MAE	RMSE	P95
Speed (km/h)	4994	+0.12	1.04	1.50	3.16
Time headway (s)	4407	+0.004	0.057	0.135	0.168
Spacing (m)	4224	+0.07	1.14	2.15	3.50

**Table 7:** Results of YOLO11-m + BoT-SORT by recording condition (test videos only).

			Speed			THW	F1
Subset	Condition	Videos	n	MAE (km/h)	In Tol. (%)	MAE (s)	THW < 1.8 s
01	Good quality	3	866	0.77	95.3	0.057	0.840
02	Strong lighting	6	1740	1.02	94.1	0.051	0.863
03	Noise	2	816	0.85	94.5	0.060	0.870
04	Noise and rain	2	826	1.66	70.7	0.065	0.800
05	Lighting, motion blur	1	746	0.90	94.9	0.058	0.845

**Table 8:** Violation detection by criterion for YOLO11-m + BoT-SORT, summed over the 14 UTFPR test videos. Ref.: number of reference violations (TP + FN).

Criterion	Ref.	TP	FP	FN	Precision	Recall	F1
Half-speed rule	1806	1676	646	130	0.722	0.928	0.812
THW<1.0 s	317	244	266	73	0.478	0.770	0.590
THW<1.8 s	1855	1691	442	164	0.793	0.912	0.848
THW<2.0 s	2222	2055	467	167	0.815	0.925	0.866
d<10 m	152	115	402	37	0.222	0.757	0.344
d<20 m	1892	1695	639	197	0.726	0.896	0.802
TTC<3 s ^1^	4	2	227	2	0.009	0.500	0.017
TTC<4 s ^1^	34	20	255	14	0.073	0.588	0.129

^1^ Too few reference violations for a meaningful evaluation.

**Table 9:** Automated decisions for the THW<1.8 s criterion (YOLO11-m + BoT-SORT, 14 UTFPR test videos, 1855 reference violations). Followers with $p_{f}\geq 0.9$ are flagged, followers with $p_{f}\leq 0.1$ are cleared and the others are routed to an operator.

Decision Rule	Flagged	Precision	Recall	To Operator	Ref. Viol. to Operator	Ref. Viol. Cleared
Hard threshold, ${\mathrm{THW}}_{f}<1.8$ s	2133	0.793	0.912	–	–	–
$p_{f}\geq 0.9$, E from calibration video	1996	0.797	0.858	246 (4.5%)	130	39
$p_{f}\geq 0.9$, E leave-one-video-out	1996	0.798	0.858	266 (4.8%)	131	37
$p_{f}\geq 0.9$, E from test video ^1^	1993	0.797	0.857	267 (4.9%)	134	37

^1^ Uses the ground truth of the test video itself; shown for comparison only.

**Table 10:** Vehicle detection on the 40 UA-DETRAC test sequences (COCO weights, no fine-tuning).

Detector	AP@0.5	AP@0.5:0.95	Precision	Recall	F1	Params (M)	GFLOPs
YOLO11-m	0.837	**0.622**	0.838	0.820	0.829	20.1	68.6
YOLO11-s	**0.842**	0.617	0.836	0.822	0.829	9.5	21.8

**Table 11:** Multi-object tracking on the 40 UA-DETRAC test sequences. IDSW: identity switches; Frag: fragmentations; MT/ML: mostly tracked/mostly lost trajectories.

Configuration	HOTA	DetA	AssA	MOTA	MOTP	IDF1	IDSW	Frag	MT	ML	ms/Frame
YOLO11-m + ByteTrack	0.664	0.610	0.728	0.671	0.855	0.785	998	4716	1666	98	19.4
YOLO11-m + BoT-SORT	**0.681**	**0.627**	**0.745**	**0.684**	0.870	**0.790**	**662**	4252	**1739**	93	22.4
YOLO11-m + OC-SORT	0.667	0.622	0.721	0.682	**0.872**	0.773	1853	**4112**	1649	116	22.9
YOLO11-s + ByteTrack	0.656	0.610	0.710	0.675	0.852	0.773	1297	5219	1640	**86**	19.7

**Table 12:** Detection and tracking on UA-DETRAC by weather condition (visual labels; sequences: 10 sunny, 9 cloudy, 9 rainy, 12 night).

Configuration	Metric	Sunny	Cloudy	Rainy	Night
YOLO11-m	AP@0.5/AP@0.5:0.95	0.840/0.594	0.846/0.632	0.799/0.594	0.875/0.661
YOLO11-s	AP@0.5/AP@0.5:0.95	0.847/0.599	0.852/0.633	0.802/0.585	0.876/0.650
YOLO11-m + ByteTrack	HOTA/IDF1/IDSW	0.652/0.815/106	0.667/0.790/194	0.639/0.754/381	0.698/0.804/317
YOLO11-m + BoT-SORT	HOTA/IDF1/IDSW	0.676/0.826/71	0.683/0.796/141	0.653/0.755/206	0.715/0.807/244
YOLO11-m + OC-SORT	HOTA/IDF1/IDSW	0.663/0.808/281	0.665/0.777/427	0.635/0.734/728	0.711/0.800/417
YOLO11-s + ByteTrack	HOTA/IDF1/IDSW	0.646/0.804/145	0.658/0.777/261	0.634/0.746/459	0.685/0.786/432

## Data Availability

The UTFPR vehicle-speed dataset [8] and the UA-DETRAC benchmark [9] used in this study are publicly available. The derived measurements and evaluation results are available from the corresponding author upon reasonable request.

## References

[1] World Health Organization (2026). Road Traffic Injuries. https://www.who.int/news-room/fact-sheets/detail/road-traffic-injuries.

[2] Kujala T., Sarkar A. (2024). Spare visual capacity and driver inattention in dynamic car following scenarios. Transp. Res. Part F Traffic Psychol. Behav..

[3] Vogel K. (2003). A comparison of headway and time to collision as safety indicators. Accid. Anal. Prev..

[4] Shaer A., Fielbaum A., Levinson D. (2026). Detecting dangerous driving via computer vision: Linking video-based indicators to road crashes. J. Transp. Saf. Secur..

[5] Hartley R., Zisserman A. (2004). Multiple View Geometry in Computer Vision.

[6] Fernández Llorca D., Hernández Martínez A., García Daza I. (2021). Vision-based vehicle speed estimation: A survey. IET Intell. Transp. Syst..

[7] Jocher G., Qiu J. (2024). Ultralytics YOLO11, Version 11.0.0. https://github.com/ultralytics/ultralytics.

[8] Luvizon D.C., Nassu B.T., Minetto R. (2017). A Video-Based System for Vehicle Speed Measurement in Urban Roadways. IEEE Trans. Intell. Transp. Syst..

[9] Wen L., Du D., Cai Z., Lei Z., Chang M.C., Qi H., Lim J., Yang M.H., Lyu S. (2020). UA-DETRAC: A new benchmark and protocol for multi-object detection and tracking. Comput. Vis. Image Underst..

[10] Bewley A., Ge Z., Ott L., Ramos F., Upcroft B. Simple online and realtime tracking. Proceedings of the 2016 IEEE International Conference on Image Processing (ICIP).

[11] Wojke N., Bewley A., Paulus D. Simple online and realtime tracking with a deep association metric. Proceedings of the 2017 IEEE International Conference on Image Processing (ICIP).

[12] Zhang Y., Sun P., Jiang Y., Yu D., Weng F., Yuan Z., Luo P., Liu W., Wang X. (2022). ByteTrack: Multi-Object Tracking by Associating Every Detection Box. arXiv.

[13] Aharon N., Orfaig R., Bobrovsky B.Z. (2022). BoT-SORT: Robust Associations Multi-Pedestrian Tracking. arXiv.

[14] Cao J., Pang J., Weng X., Khirodkar R., Kitani K. Observation-Centric SORT: Rethinking SORT for Robust Multi-Object Tracking. Proceedings of the 2023 IEEE/CVF Conference on Computer Vision and Pattern Recognition (CVPR).

[15] Bernardin K., Stiefelhagen R. (2008). Evaluating multiple object tracking performance: The CLEAR MOT metrics. EURASIP J. Image Video Process..

[16] Ristani E., Solera F., Zou R., Cucchiara R., Tomasi C., Hua G., Jégou H. (2016). Performance Measures and a Data Set for Multi-target, Multi-camera Tracking. Proceedings of the Computer Vision—ECCV 2016 Workshops.

[17] Luiten J., Osep A., Dendorfer P., Torr P., Geiger A., Leal-Taixé L., Leibe B. (2020). HOTA: A Higher Order Metric for Evaluating Multi-object Tracking. Int. J. Comput. Vis..

[18] Dubská M., Herout A., Juránek R., Sochor J. (2015). Fully Automatic Roadside Camera Calibration for Traffic Surveillance. IEEE Trans. Intell. Transp. Syst..

[19] Revaud J., Humenberger M. Robust Automatic Monocular Vehicle Speed Estimation for Traffic Surveillance. Proceedings of the 2021 IEEE/CVF International Conference on Computer Vision (ICCV).

[20] Sochor J., Juránek R., Špaňhel J., Maršík L., Široký A., Herout A., Zemčík P. (2019). Comprehensive Data Set for Automatic Single Camera Visual Speed Measurement. IEEE Trans. Intell. Transp. Syst..

[21] Krajewski R., Bock J., Kloeker L., Eckstein L. (2018). The highD Dataset: A Drone Dataset of Naturalistic Vehicle Trajectories on German Highways for Validation of Highly Automated Driving Systems. arXiv.

[22] Charouh Z., Ezzouhri A., Ghogho M., Guennoun Z. Headway and Following Distance Estimation using a Monocular Camera and Deep Learning. Proceedings of the 13th International Conference on Agents and Artificial Intelligence (ICAART).

[23] St-Aubin P., Saunier N., Miranda-Moreno L. (2015). Large-scale automated proactive road safety analysis using video data. Transp. Res. Part C Emerg. Technol..

[24] Hayward J.C. (1972). Near-miss determination through use of a scale of danger. Highw. Res. Rec..

[25] Mahmud S.S., Ferreira L., Hoque M.S., Tavassoli A. (2017). Application of proximal surrogate indicators for safety evaluation: A review of recent developments and research needs. IATSS Res..

[26] Republic of Türkiye (1997). Karayolları Trafik Yönetmeliği (Highway Traffic Regulation), Article 101. Resmî Gazete.

